# HSP70 Promoter-Driven Activation of Gene Expression for Immunotherapy Using Gold Nanorods and Near Infrared Light

**DOI:** 10.3390/vaccines2020216

**Published:** 2014-03-25

**Authors:** Helen A. Andersson, Yoo-Shin Kim, Brian E. O’Neill, Zheng-Zheng Shi, Rita E. Serda

**Affiliations:** 1 Department of Nanomedicine, Houston Methodist Research Institute, Houston, TX 77030, USA; E-Mail: handersson@sbcglobal.net; 2 Department of Translational Imaging, Houston Methodist Research Institute, Houston, TX 77030, USA; E-Mails: ykim@houstonmethodist.org (Y.-S.K.); beoneill@houstonmethodist.org (B.E.O.); zshi@houstonmethodist.org (Z.-Z.S.); 3 Michael E. DeBakey Department of Surgery, Baylor College of Medicine, Houston, TX 77030, USA; E-Mail: rita.serda@bcm.edu

**Keywords:** gold nanorod, HSP70 promoter, near infrared, immunotherapy, cancer

## Abstract

Modulation of the cytokine milieu is one approach for vaccine development. However, therapy with pro-inflammatory cytokines, such as IL-12, is limited in practice due to adverse systemic effects. Spatially-restricted gene expression circumvents this problem by enabling localized amplification. Intracellular co-delivery of gold nanorods (AuNR) and a heat shock protein 70 (HSP70) promoter-driven expression vector enables gene expression in response to near infrared (NIR) light. AuNRs absorb the light, convert it into heat and thereby stimulate photothermal expression of the cytokine. As proof-of-concept, human HeLa and murine B16 cancer cells were transfected with a HSP70-Enhanced Green Fluorescent Protein (EGFP) plasmid and polyethylenimine (PEI)-conjugated AuNRs. Exposure to either 42 °C heat-shock or NIR light induced significant expression of the reporter gene. *In vivo* NIR driven expression of the reporter gene was confirmed at 6 and 24 h in mice bearing B16 melanoma tumors using *in vivo* imaging and flow-cytometric analysis. Overall, we demonstrate a novel opportunity for site-directed, heat-inducible expression of a gene based upon the NIR-absorbing properties of AuNRs and a HSP70 promoter-driven expression vector.

## 1. Introduction

In addition to malignant cells, tumors consist of non-transformed host cells, such as fibroblasts and immune cells, vascular tissue, cytokines, and the surrounding extracellular matrix. The tumor microenvironment is a complex interrelated system that plays an important role in malignant cell survival, growth, proliferation, and metastasis. Cancer treatments have historically only targeted the malignant cell itself, and as a result, they rarely prevent recurrence of disease or progression of metastasis [1]. The importance of the tumor microenvironment and the interactions between the different cell types and components has become increasingly recognized. The balance of cellular and cytokine interactions and signaling within this milieu has a major impact on whether the tumor mass regresses or grows, and whether the malignant cells remain localized or metastasize to distant sites. Effective eradication of malignant disease requires therapeutic strategies that focus on the whole tumor as well as metastatic tissue.

Gold nanoparticles (AuNP) and nanorods (AuNR) have emerged as attractive nanomaterials for biological and biomedical applications because of their physical and chemical properties. The particles absorb and scatter visible and near-infrared (NIR) light upon excitation of their surface plasmon resonance (SPR) oscillation, which can be tuned over a wide spectral range by changing intrinsic particle parameters such as size and shape [2]. Rod-shaped gold particles have shown promise over spherical shaped AuNP due to fact that they display two separate SPR bands corresponding to their width and length, allowing their longitudinal plasmon bands to range from the visible (600 nm) to the near infrared (1100 nm and up) regions of the electromagnetic spectrum [3]. Their ability to absorb light in the near infrared region, and subsequent conversion of the applied energy into heat, has led to the use of AuNPs and AuNRs for hyperthermia-based applications, including cancer therapy. Photothermal ablation of solid tumors has been investigated in various preclinical models and is currently being evaluated in the clinic [4,5,6]. Nanoparticles can be delivered to the tumor either passively, accumulating in the tumor through the enhanced permeability and retention effect (EPR), or actively targeted to receptors on the tumor or tumor-associated vasculature [7,8,9,10]. The AuNR are easily functionalized with peptides, proteins, antibodies, or nucleic acids for targeting or for creating multifunctional platforms for therapeutic and diagnostic purposes [11,12]. Clinical advantages of using gold nanoparticles include easy synthesis, targeting capability, high biocompatibility, cost effectiveness, and easy clearance from the body [13,14]. They have an excellent track record of being well tolerated in humans and are currently in the process of obtaining FDA approval for clinical use [15,16].

Several strategies that allow control of both spatial and temporal expression of transgenes have been developed. Spatial resolution is often achieved by the use of tissue- or cell-specific promoters, or exploitation of the heat-shock response, a temperature-sensitive defense mechanism [17,18,19]. The heat-shock response is mediated by a transcription factor known as heat shock factor (HSF). HSF is synthesized constitutively, but remains dormant under normal conditions. In response to heat, HSF trimerizes and binds with high affinity to heat shock promoters containing specific binding elements, leading to the transcription of heat-shock proteins [20]. Temporal resolution may be under the control of external cues, such as NIR laser light. Induction of heat shock promoter (HSP)-mediated gene expression by laser light is a promising approach for achieving temporal and spatial control of gene expression [21,22]. Other approaches beyond NIR light have considerable technical limitations related to their use of UV, short-wavelength visible (vis), and infrared (IR) laser light, due to poor penetration into biological tissue. Conversely, biological tissue is relatively transparent to light inside the diagnostic window of 700–1100 nm [23]. NIR laser light has been shown to penetrate 10 cm through breast tissue or 4 cm through deep muscle [24]. The ability of nanorods to absorb NIR light makes them particularly well-suited to biomedical applications since the absorbance of the surrounding tissue in this region is low, allowing for minimally invasive delivery of energy to tumor cells that have taken up the AuNR, without inducing damage to intervening and surrounding normal tissue.

**Figure 1:**
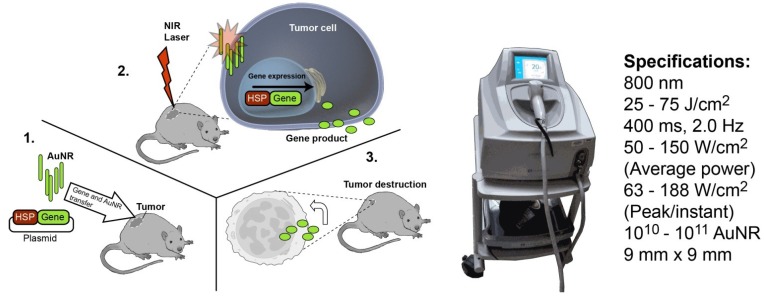
Schematic showing near infrared (NIR)-driven hyperthermia mediated therapy. (1) AuNRs complexed with therapeutic HSP70B promoter-driven gene vectors will be injected into mice intratumorally or intravenously with targeting moieties; (2) NIR laser treatment of the tumor site will result in localized heating of the AuNR, which in turn will induce expression of the therapeutic gene; (3) The predicted end result is tumor growth inhibition and tumor mass reduction. The Light Sheer ET Lumenis FDA-approved NIR laser light source and machine specifications are shown to the right.

While cytokine and drug therapeutics are effective against cancer cells, they also cause systemic effects and damage to healthy tissue. To accurately regulate the levels of therapeutic gene expression to achieve enhanced efficacy and minimal toxicity, we are proposing to drive the expression of target genes using a heat-inducible promoter. Our proposed vector system consists of heat generating AuNRs and a therapeutic gene expression vector under the control of the human heat shock promoter 70 (HSP70). We hypothesize that exposure of pathological tissue to a near infrared (NIR) laser source will cause the AuNR to absorb the NIR light and convert it to heat, thus inducing spatially confined, photo-thermal expression of the target gene (Figure 1). Controlled gene expression within the tumor microenvironment could include expression of cytokines, such as IL-12 or interferon gamma, leading to tumor infiltration and activation of immune cells, including antigen presenting cells, natural killer (NK) cells, type 1 helper T cells, and cytotoxic lymphocytes (CTL), resulting in immune targeting of the diseased cells. Alternatively, expression of immuno-suppressive molecules or cytokines, such as IL-10, could promote tissue transplantation, or suicide genes could lead to apoptosis of targeted cells.

Wei *et al.* [25] demonstrated HSP70B-driven expression of 1L-12 using adenovirus. Mice with subcutaneous Hep3B tumors were given an intratumoral injection of adenovirus encoding both heat inducible IL-12 and constitutively expressed granulocyte macrophage colony stimulating factor (GM-CSF). Using external heating of the limb with a water bath, they demonstrated elevated IL-12 levels during 3 separate heating events. While effective, this technique requires whole limb heating. We propose a method to achieve selective heating of diseased or immune cells using non-invasive NIR light and delivery of AuNR to cells of interest.

Herein we describe an adjuvant method in which NIR induced hyperthermia is mediated by the cellular loading of nanorods and monitored by the expression of a HSP70 driven reporter within the same cell. Preliminary efficacy studies are presented in nude mice bearing orthotopic B16 melanoma tumors. Target tumor cells are transfected *ex vivo* with the reporter plasmid and AuNRs prior to transplantation and NIR exposure. We have optimized AuNR-loading into tumor cells and induction of gene expression using a NIR dose sufficient to induce GFP reporter expression, yet low enough to maintain cell viability.

## 2. Experimental

### 2.1. Materials

Gold nanorods (AuNR) conjugated to polyethyleneimine (PEI) were purchased from Nanopartz™ Inc., Loveland, CO. The 800 nm NIR light source was an FDA approved clinical diode laser device obtained from Lumenis, Inc. (Lightsheer ET, Lumenis, Inc., Santa Clara, CA, USA) with peak power of 1600 W, laser fluence 10–100 J/cm^2^ (Figure 1). B16F10-luc melanoma cells, stably transfected with the firefly luciferase gene, were purchased from Caliper (Perkin Elmer, Waltham, MA, USA).

### 2.2. Cloning of HSP70 Promoter-Driven GFP Reporter

The 400 bp minimal human HSP70B promoter fused with the EGFP gene, a kind gift from Dr. Chrit Moonen of Université Victor Ségalen, France, [26] was cloned into the pGL3 vector (Promega, Madison, WI, USA). The construct was confirmed by restriction digestion, and the reporter expression was verified through transfection of HeLa cells as described below.

### 2.3. Verification of in Vitro GFP Expression in Cells and Uptake of AuNRs

HeLa or B16 cells were transfected with HSP70-pGL3 using Lipofectamine LTX reagent (Invitrogen, Grand Island, NY, USA) at a ratio of 1:4 DNA:lipofectamine, and 24 h later the cells were either left at 37 °C or heat-shocked at 42.5 °C for 30 min. Lipofectamine LTX was chosen to avoid activation of the HSP promoter shown to occur with other transfection reagents [27]. The following day, cells were analyzed for EGFP expression using a LSR Fortessa flow cytometer (Becton Dickinson), or by fluorescence microscopy using a Nikon A1 confocal microscope.

B16F10-luc cells were plated in 96-well plates and AuNR were added at increasing concentrations. After 24 h, the cells were washed and lysed. The number of AuNR in each well was determined using UV/VIS spectroscopy. A standard curve was generated by adding serial dilutions of AuNR with known concentration to wells containing saline and cell lysate.

### 2.4. In Vitro Optimization of NIR-Induced Expression

To investigate the effect of NIR laser radiation on the induction of transgene expression, we loaded polyethyleneimine (PEI)-conjugated gold rods (10 nm transverse diameter with surface plasmon resonance (SPR) peak of 808 nm) with the HSP70-EGFP vector in the presence of Lipofectamine LTX and incubated HeLa cells with the complexes overnight. The following day, the cells were exposed to varying laser fluencies (25–75 J/cm^2^) at 10 or 20 pulses using the 800 nm Lumenis laser. Twenty four hours after NIR treatment, the cells were analyzed for EGFP expression and viability by flow cytometric analysis.

### 2.5. Optimization of in Vivo NIR-Induced GFP Expression

B16F10-luc melanoma cells were transiently transfected with the HSP70-EGFP plasmid using Lipofectamine LTX, followed 6 h later by addition of PEI-AuNRs at concentrations of 10^12^ overnight. Cells were injected subcutaneously into the flanks of nude mice. NIR irradiation at varying fluencies was applied to the nascent tumors. *In vivo* fluorescent and bioluminescent imaging (BLI) was performed at various time points to monitor EGFP and luciferase expression. Excised tumors were analyzed by flow cytometry or fluorescent microscopy for GFP expression.

To verify that we could successfully observe heat-shock induced GFP expression *in vivo*, we first established, as a positive control, B16F10-luc cell lines carrying HSP70-EGFP and AuNPs exposed to heat-shock *in vitro* prior to being injected into the animal. Heat-shocked cells were exposed to 42 °C in a water bath for 30 min. Negative controls received a sham treatment at 37 °C.

To verify *in vivo* NIR induced gene expression, B16 cells were transfected as above. 2 × 10^7^ tumor cells were injected into the flanks of nude mice the following day and treated with NIR laser within the first hour, initially using laser parameters established *in vitro.* The experiment was repeated a second time with lower cell numbers, fluencies and duty cycles. Expression of GFP and luciferase in the tumors at 6 and 24 h after injection was tested via *in vivo* imaging and flow-cytometric analysis.

## 3. Results and Discussion

### 3.1. Verification of Transfection and Uptake of AuNPs

**Figure 2:**
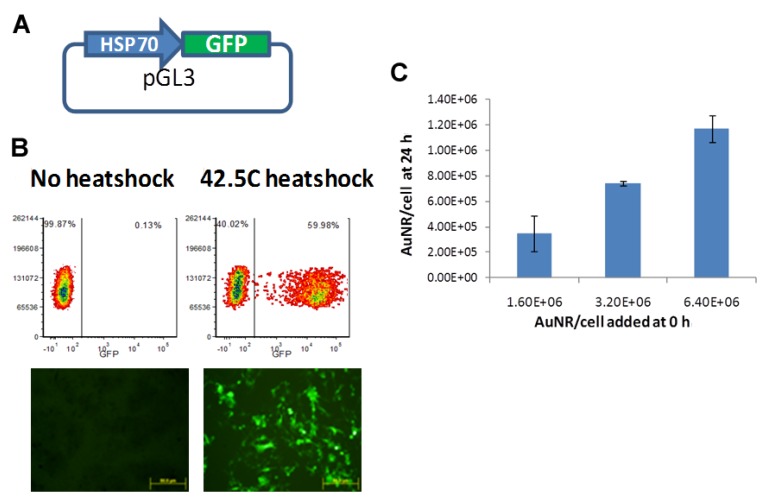
Transfection of HeLa cells with the HSP70- Enhanced Green Fluorescent Protein (EGFP) expression vector and quantitative measurement of AuNR uptake *in vitro*. (**A**) Cartoon of the expression vector; (**B**,**C**) HeLa cells were transfected with the vector using lipofectamine LTX reagent, and 24 h later the cells were either left at 37 °C or heat-shocked at 42.5 °C for 30 min. The following day, cells were analyzed for EGFP expression via flow cytometric analysis or fluorescence microscopy (**B**); B16-luc cells were plated in 96-well plates and AuNR were added at increasing concentrations. After 24 h, the cells were washed and lysed. The number of AuNR in each well was determined using UV/VIS spectroscopy (**C**); A standard curve was generated by adding known numbers of AuNR to wells, and a graph showing the number of particles present in the cells at 24 h *vs.* the number added at 0 h is presented.

As shown in Figure 2, 60% of HSP70-EGFP transfected B16 cells showed an increase in EGFP expression following heat-shock compared to <1% of transfected cells without heat-shock. This negligible activity in the “off” state confirms that gene expression from the HSP70 promoter is tightly regulated. Figure 2C shows high uptake of the PEI-coated AuNPs during incubation, with 20%‑25% of AuNPs in solution ending up in the cells.

### 3.2. In Vitro Optimization of NIR-Driven Expression

**Figure 3:**
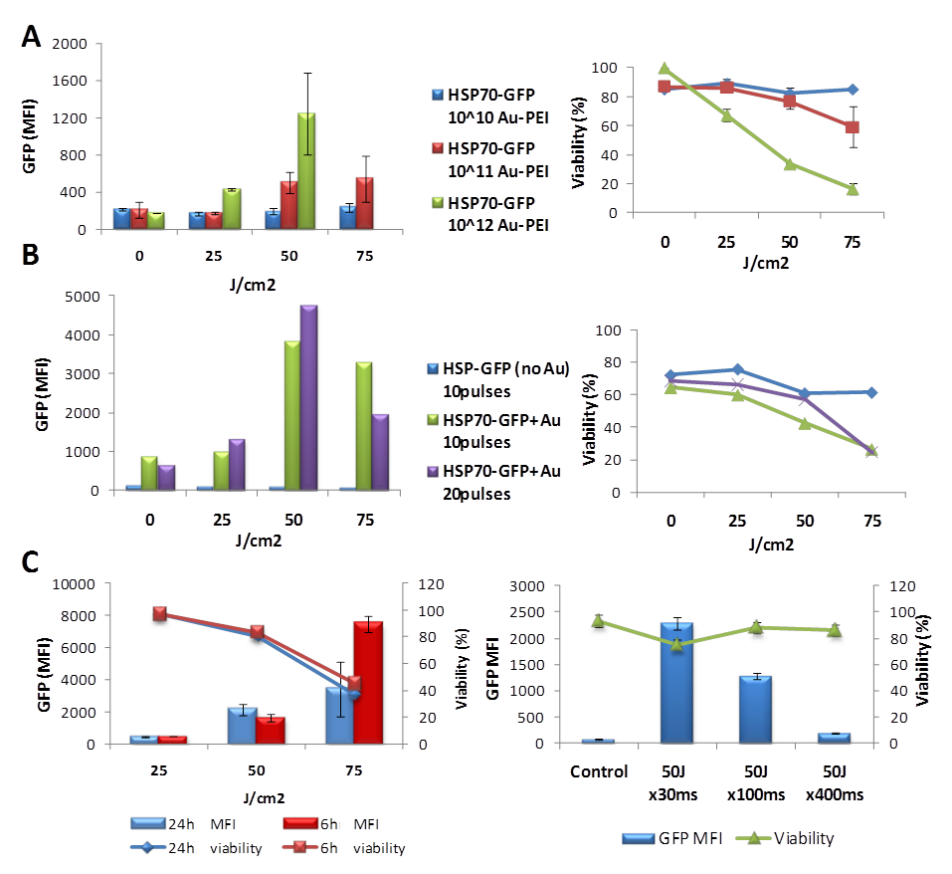
Cell viability and GFP expression after *in vitro* NIR treatment. (**A**) B16-Luc cells (1 × 10^5^ per well in 24-well glass bottom plates) were incubated with HSP70-GFP and 10^1^^0^, 10^11^, or 10^12^ AuNR per well. The following day, the cells were treated with NIR laser with 10 pulses at 0, 25, 50, or 75 J/cm^2^ (**B**) B16-Luc cells were incubated overnight with or without 10^11^ AuNR per well and treated with NIR laser with 10 or 20 pulses. Cells were analyzed for GFP expression and viability by flow cytometery the following day; (**C**) Mean GFP fluorescence intensity (MFI) and percent viable cells (measured by uptake of propidium iodide) as a function of laser fluence (25, 50 and 75 J/cm^2^; left), time after NIR treatment (6 h or 24 h; left) and pulse duration (30, 100, or 400 ms; right).

The results of the *in vitro* NIR optimization are shown in Figure 3. We found that the optimal dose to reach the highest level of EGFP expression with the lowest cell death/toxicity is achieved by using approximately 10^11^ AuNR and 10 pulses at 50 J/cm^2^, with a 30 ms pulse length. In general, cells are driven to increase production of EGFP with increased heating up to some threshold, at which point cell damage rapidly reduces expression and eventually viability. Heating efficiency increases as expected with increased loading of AuNPs (Figure 3A) and pulse numbers (Figure 3B). At a laser fluence of 25 J/cm^2^ relatively little gene expression occurs, with increases in fluence to 50 J/cm^2^ inducing increased expression and relatively high viability (approximately 80%). While gene expression was highest at 75 J/cm^2^, cell viability dropped to 44% and 37% at 6 h and 24 h, respectively. Increasing the length of the pulses from 30 ms to 400 ms actually decreases the GFP expression (Figure 3C; right), demonstrating that peak power is more important than total power. In Figure 3C, the peak power is decreasing (the same energy, 50 J, is spread over a longer pulse), even though the total power is the same. In general, some loss of viability appears as a necessary price for high expression.

### 3.3. In Vivo HSP-Promoter-Driven Expression

**Figure 4:**
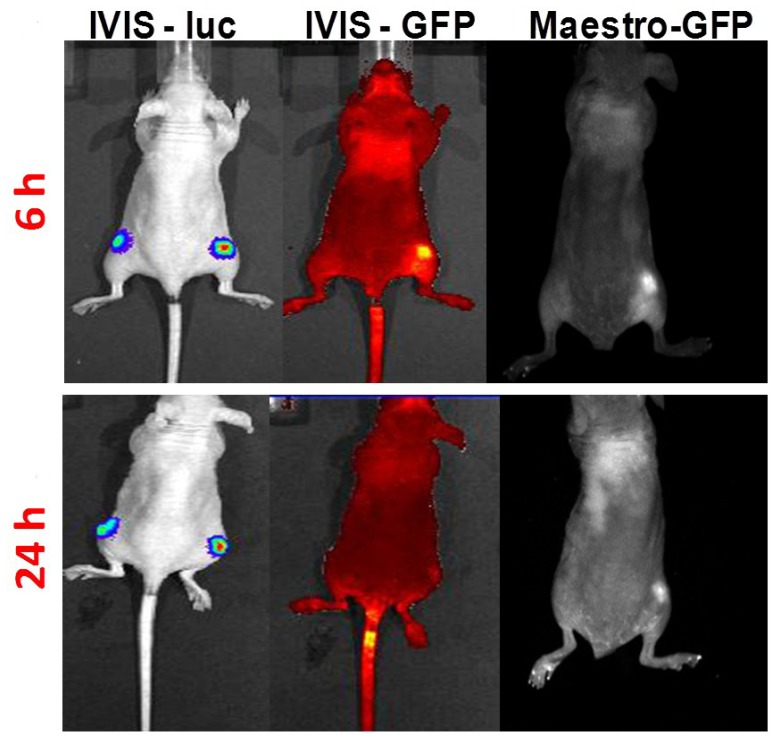
*In vivo* validation studies. B16-luc cells were transfected with the HSP-GFP vector using lipofectamine LTX reagent (Invitrogen), and 24 h later the cells were either left at 37 °C or heat-shocked at 42.5 °C for 30 min in a water bath. The following day, cells were analyzed for EGFP expression via flow cytometry or fluorescence microscopy. Transfected B16 cells either exposed to heat-shock or kept at 37 °C were injected into flanks of nude mice and imaged at 6 h and 24 h for luciferase expression (using the IVIS imaging system) and GFP (using the IVIS and Maestro imaging systems). Mice were injected with D-Luciferin before imaging. The right leg of the mouse was injected with heat-shocked cells whereas the cells injected into the left leg were left untreated.

Figure 4 shows animals inoculated with heat-shocked cells on the right and sham-heated cells on the left. Both sets of B16F10-luc cells show up well under BLI, but only the heated cells are detected under fluorescent imaging. The signal from the positive controls is strong enough to be detected through the skin of the *in vivo* by both the IVIS200 and Maestro imaging using FITC filters.

**Figure 5:**
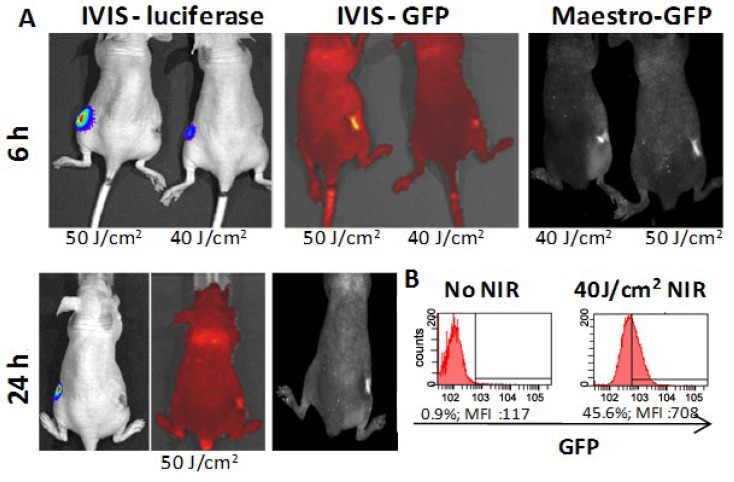
*In vivo* imaging of luciferase and GFP expression after NIR laser treatment. (**A**) B16-luciferase cells were transfected with the HSP70-GFP vector 24 h before NIR treatment and AuNR (10^6^/cell) were added 12 h after transfection. Cells were washed before being harvested and 2 × 10^7^ cells were injected in each flank of two nude mice. Immediately following injection, the right flank of each mouse was treated with NIR laser for 10 pulses at 30 ms at either 40 or 50 J/cm^2^. The left flank was left non-treated. Mice were imaged at 6 h via IVIS and Maestro *in vivo* imaging systems. At 24 h after treatment, one mouse was imaged as just described, and the other mouse was sacrificed and the tumor analyzed for GFP expression via flow cytometry analysis (**B**).

As shown in Figure 5, strong EGFP expression could be detected 6 hours after NIR treatment at both 40 J/cm^2^ and 50 J/cm^2^ laser at 30 ms pulse length. This was confirmed with FACS on the 40 J/cm^2^ and was confirmed to last at least 24 h in the 50 J/cm^2^ animal. The laser treatment at the higher power seemed to result in more physical damage to the skin of the animal. Bioluminescent imaging (BLI) was also used to monitor luciferase expression in the cells with IVIS200. Interestingly, the luciferase signal was intact on the non-treated left flanks in both mice, but suppressed on the NIR treated right flanks. This finding is in agreement with previous studies reporting that luciferase is highly sensitive to temperature, and thus can function as an indicator of a successful thermal treatment [28]. Further experiments indicated that detectable expression may be achieved with half the gold loading, and using 5 instead of 10 pulses, still at 40 J/cm^2^. Reducing the laser fluence to 30 J/cm^2^ or below resulted in no observable expression. Treatment with laser of cells without AuNPs did not result in any increase in EGFP expression or loss of bioluminescence.

## 4. Conclusions

Although gene therapy has shown great promise both for cancer and infectious diseases, the major challenge lies in the development of safe and effective delivery systems that can lead to controlled expression of therapeutics. Despite the high transduction efficiency and long-term gene expression of viral vectors, the complexity of their manufacturing and *in vivo* safety issues remains hurdles to be overcome. We demonstrate a novel opportunity for site-directed, heat-inducible gene expression based upon the NIR-absorbing properties of AuNRs. We have optimized AuNR-loading into tumor cells and induction of gene expression using a NIR dose sufficient to induce GFP reporter expression, yet low enough to maintain cell viability. We have also confirmed induction of gene expression by NIR laser in an *in vivo* tumor model.

Future studies will include delivery of therapeutic genes with heat sensitive promoters using viral nanoparticles (NPs) to prolong the presence of the gene in cells, permitting repeat heating cycles and temporal control of gene expression without the need for continuous gene delivery or *ex vivo* transfection of cells with the HSP70B promoter plasmid. Simultaneous accumulation of viral NPs and AuNRs or carbon-based NPs in the tumor will be achieved using either targeting ligands or by selective heating of the tumor through non-invasive tissue heating, such as radiofrequency (RF) wave induced hyperthermia which supports increased intratumoral blood flow and nanoparticle accumulation [29]. Tumor tissue is highly susceptible to selective RF-induced heating based on its unique biological composition [30], enabling the use of RF energy to drive gene expression or to promote tumor necrosis. Thus RF-driven hyperthermia is currently being explored as a mechanism for achieving both NP accumulation and for tumor ablation. While NIR light is ideal for treating melanoma or near-surface tumors, RF complements this technique by enabling deep tissue penetration.
